# Co-repressors AtSDR4L and DIG1 interact with transcription factor VAL2 and promote Arabidopsis seed-to-seedling transition

**DOI:** 10.1093/plphys/kiae225

**Published:** 2024-04-23

**Authors:** Bailan Lu, Milad Alizadeh, Ryan Hoy, Renwei Zheng, Dongeun Go, Liang Song

**Affiliations:** Department of Botany, University of British Columbia, Vancouver, British Columbia V6T 1Z4, Canada; Department of Botany, University of British Columbia, Vancouver, British Columbia V6T 1Z4, Canada; Department of Botany, University of British Columbia, Vancouver, British Columbia V6T 1Z4, Canada; Department of Botany, University of British Columbia, Vancouver, British Columbia V6T 1Z4, Canada; Department of Botany, University of British Columbia, Vancouver, British Columbia V6T 1Z4, Canada; Department of Botany, University of British Columbia, Vancouver, British Columbia V6T 1Z4, Canada

## Abstract

Two transcriptional co-repressors physically interact with a transcription factor that is known to recruit a multi-protein complex, which promotes the repression of seed maturation genes by depositing trimethylation marks on lysine 27 of the histone 3 tails.

Dear Editor,

Robust seedling establishment requires transcriptional repression of seed maturation genes, often through the epigenetic modification of histones by the Polycomb Repressive Complexes (PRCs). The core components of PRCs are moderately conserved between animals and plants, while many PRC-associated proteins rendering regulatory specificity are more diverse (Xiao and Wagner 2015). *Seed Dormancy 4* (*Sdr4*) is a quantitative trait locus in rice (*Oryza sativa*) that represses preharvest sprouting (Sugimoto et al. 2010; Chen et al. 2021; Zhao et al. 2022). *ARABIDOPSIS THALIANA SEED DORMANCY 4-LIKE* (*AtSDR4L*), also known as *SEED DORMANCY FOUR-LIKE 1* (*SFL1*)/*REVERSAL OF RDO5 1* (*ODR1*), is an *Sdr4* ortholog in Arabidopsis (*Arabidopsis thaliana*) (Cao et al. 2020; Liu et al. 2020; Wu et al. 2022; Zheng et al. 2022). *Atsdr4l* loss-of-function mutants phenocopy the *prc* mutants, displaying delayed seed germination and abnormal seedling establishment (Wu et al. 2022; Zheng et al. 2022). AtSDR4L and its Arabidopsis paralogs, collectively known as *Dynamic Influencer of Gene Expression* (*DIG*)/*ABA-INDUCED TRANSCRIPTION REPRESSOR* (*AITR*)/*SFL*, are proposed to repress transcription (Song et al. 2016; Tian et al. 2017; Zheng et al. 2022). Here, we demonstrate the physical interaction and genome-wide binding similarities between AtSDR4L, DIG1, and PRC2-associated protein VIVIPAROUS1/ABI3-LIKE 2 (VAL2). We propose that AtSDR4L and its homolog likely use PRC2-mediated H3K27me3 deposition to regulate downstream targets.

## DIG1 and AtSDR4L physically interact with VAL2

**Figure 1.**
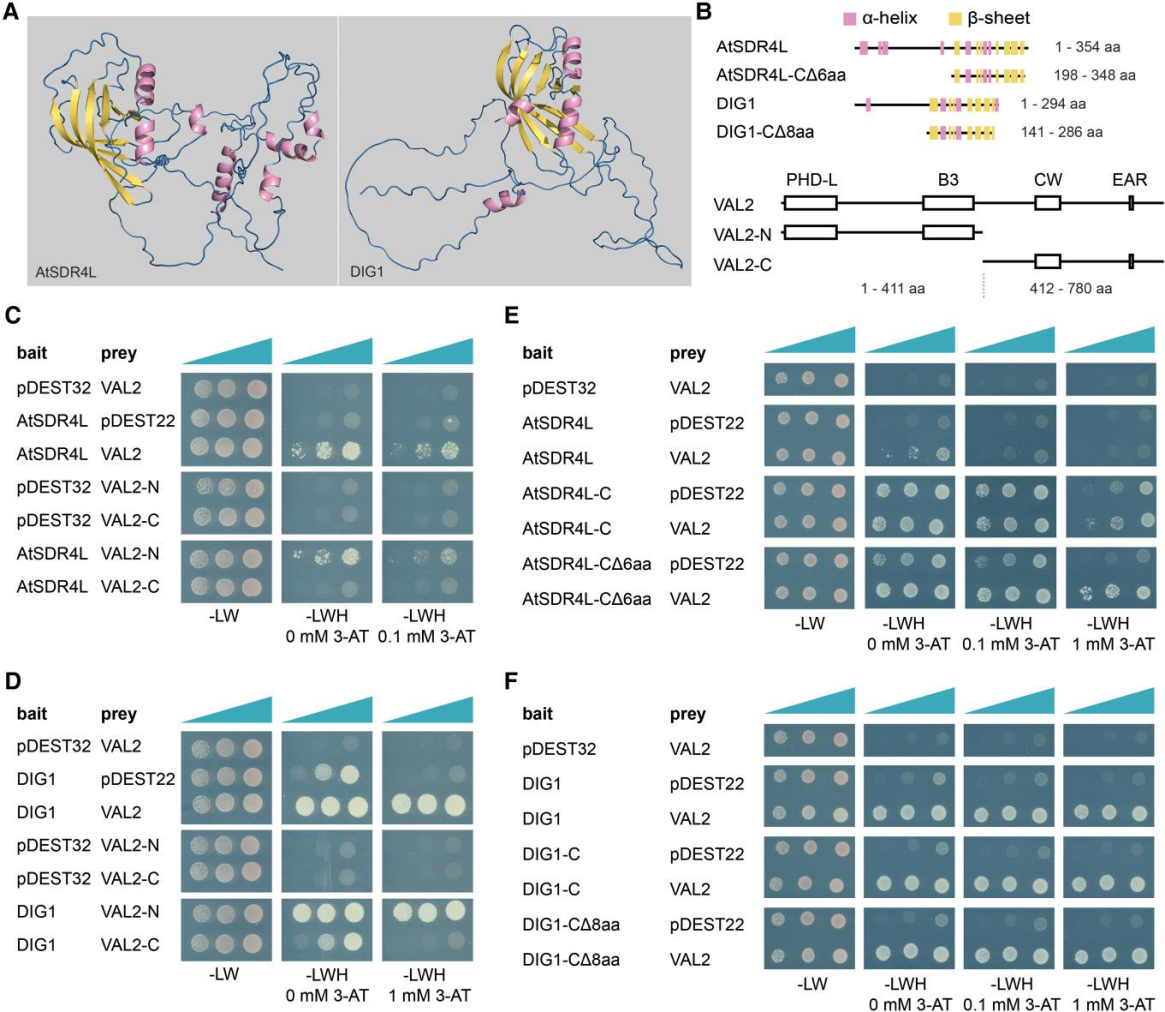
AtSDR4L and DIG1 physically interact with VAL2. **A)** Structural prediction of AtSDR4L and DIG1 by AlphaFold2 and diagrams generated by PyMOL^TM^ 2.5.7. **B)** A schematic representation of full-length and truncated AtSDR4L, DIG1, and VAL2. Alpha helices and beta sheets in AtSDR4L and DIG1 are colour-coded and correspond to the structures in AlphaFold2 prediction; -CΔ6aa and -CΔ8aa indicate removal of the last 6 and 8 amino acid (aa) residues from AtSDR4L-C and DIG1-C, respectively; plant homeodomain-like (PHD-L), B3 DNA-binding, Cys- and Trp-containing (CW) domains, and the Ethylene-responsive element binding factor-associated Amphiphilic Repression (EAR) motif in VAL2 are labeled as boxes or a vertical line. **C and D)** Yeast 2-hybrid assay using yeasts co-expressing the indicated pairs of bait and prey show that AtSDR4L **C)** and DIG1 **D)** physically interact with the N-terminal half of VAL2. Bait proteins were expressed from the pDEST32 vector that encodes a GAL4 DNA-binding domain and prey proteins were expressed from the pDEST22 vector that encodes a GAL4 DNA activation domain. Yeasts were plated on double dropout media and triple dropout media supplemented with different concentrations of 3-amino-1,2,4-triazole (3-AT), on a dilution gradient of 1:100 (v/v), 1:10 (v/v), and undiluted (shown by triangles above each lane). -L, -W, -H: dropout media deprived of leucine, tryptophan, and histidine. **E and F)** Yeast 2-hybrid assay using yeasts co-expressing the indicated pairs of bait and prey show that the C-terminal half of AtSDR4L **E)** and DIG1 **F)** physically interact with VAL2.

Based on the similarities in mutant phenotypes and differential gene expression, we examined whether AtSDR4L physically interacts with PRC core and accessory proteins. AlphaFold (Jumper et al. 2021; Mirdita et al. 2022) predicted that N-terminal halves of AtSDR4L and DIG1 are disordered, while the C-terminal halves encompass multiple α-helices and β-sheets (Fig. 1, A and B). Yeast two-hybrid showed that VAL2, but not its homolog VAL1, was the only protein that interacts with AtSDR4L, DIG1, and DIG-Like1 (DIL1) (Supplementary Fig. S1, A and B, Supplementary Table S1). Autoactivation made the interaction inconclusive for the remaining family members (Supplementary Fig. S1B). To narrow down interacting regions, VAL2 was split between the B3 DNA-binding domain and Cys- and Trp-containing (CW) domain (Fig. 1B, Supplementary Table S2). The N-terminal half of VAL2 (VAL2-N), but not the C-terminal half (VAL2-C), interacts with DIG1 and AtSDR4L (Fig. 1, C and D). Further truncation of VAL2-N abolishes the interaction with DIG1 (Supplementary Fig. S1C), suggesting that the PHD-L or B3 domain alone is not sufficient to mediate the interaction, or that the heavily truncated VAL2 fragments cannot fold properly to interact with DIG1. Truncated constructs guided by AlphaFold predictions revealed that the C-terminal fragment of DIG1 (DIG1-C) strongly interacts with VAL2 (Fig. 1F). To mitigate the autoactivation of AtSDR4L-C, we removed the last 6 and 8 residues from AtSDR4L-C and DIG1-C, respectively. The resulting AtSDR4L-CΔ6aa and DIG1-CΔ8aa fragments consistently interact with VAL2 (Fig. 1, E and F). Additionally, the interaction between DIG1 and VAL2-N was supported by bimolecular fluorescence complementation (Supplementary Fig. S1D), although poorly expressed full-length VAL2 protein in *Nicotiana benthamiana* leaves (Supplementary Fig. S1E) and relatively weaker interaction with AtSDR4L prevented us from testing the interactions extensively. Collectively, we show that the interactions depend on VAL2-N and the C-terminal halves of AtSDR4L and its homologs.

**Figure 2.**
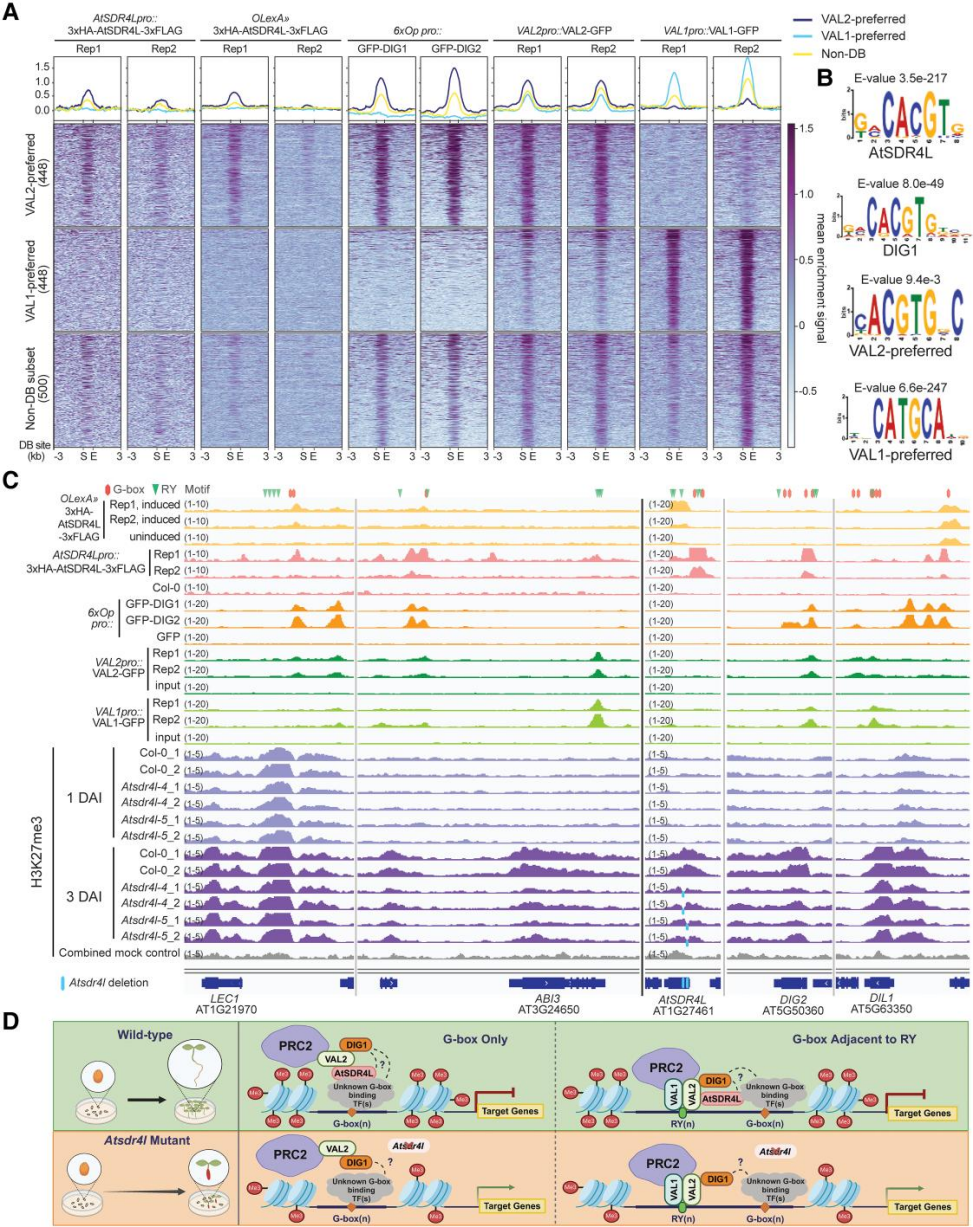
AtSDR4L and DIG1 share targets with VAL2 genome-wide. **A)** Profile plots and heatmaps representing the enrichment signal (mean normalized log2 ratio relative to respective control) of AtSDR4L, DIG1, DIG2, VAL2, and VAL1 over the binding regions preferred by VAL2 and VAL1, as well as the 500 non-differentially bound (non-DB) regions with the highest false discovery rates (FDRs) from VAL1–VAL2 DiffBind analysis. S = peak start, E = peak end. **B)** Enriched motifs (*E*-value < 0.05) identified by MEME-ChIP using ±100 bp sequences from the summits of AtSDR4L (Irreproducible Discovery Rate ≤ 0.1) and DIG1 (q < 0.05) ChIP-seq, or ±200 bp regions from VAL1 and VAL2 DiffBind summits (FDR < 0.05). Shuffled input sequences were used as background for motif enrichment analysis. **C)** Integrative Genomics Viewer (IGV) showing the co-localized binding of AtSDR4L with DIG1 and DIG2, and VAL2 in the upstream regions of *LEC1* and *ABI3* loci. AtSDR4L also binds upstream of itself, and overlaps with DIG1 and DIG2 binding sites in the regions upstream of *DIG2*, and *DIL1* genes. H3K27me3 deposition was compared among Col-0, *Atsdr4l-4*, and *Atsdr4l-5* at 1 d after imbibition (DAI) and 3 DAI. Numbers above tracks indicate normalized data range in tiled data format. Motif labels above IGV tracks: diamond represents G-box motif (CACGTG); arrowhead represents RY motif (CATGCA). Highlighted vertical lines on *AtSDR4L* gene represent deletions in *Atsdr4l-4* and *Atsdr4l-5*. **D)** A proposed model for AtSDR4L-VAL2 interaction in repressing gene expression during the seed-to-seedling phase transition. In the G-box abundant regions without full-length RY motif, the physical association between VAL2 and AtSDR4L (or DIG1) can help recruit PRC2 to target loci for histone modification. In regions where G-box is in close proximity with RY, both motifs may be involved in PRC2 recruitment. The targeting of PRC2 can be mediated by VAL1–VAL2 through their direct binding at the RY motif (Chen et al. 2020), and the indirect association of PRC2-VALs with the G-box motif can be bridged by AtSDR4L (or DIG1). Both modes of action require additional TFs that directly bind to the G-box element. These repress the expression of target genes in wild-type seedlings (vertical bar above genes). In *Atsdr4l* loss-of-function mutant, PRC2-mediated H3K27me3 deposition is attenuated, resulting in the derepression of transcriptional regulators (arrow above target genes), delayed seed germination (longer transition arrow in mutant and shorter arrow in wild-type), and recurrence of embryonic traits in seedlings (excessive neutral lipid shown in swollen hypocotyl). The extent of functional redundancy and the interaction between AtSDR4L and DIG1 remain elusive.

To examine the biological relevance of the interactions in planta, a native promoter-driven construct *AtSDR4Lpro::3xHA-AtSDR4L-3xFLAG* was introduced into *Atsdr4l-4*. Because AtSDR4L protein is strongly upregulated by abscisic acid (ABA) in young seedlings (Supplementary Fig. S2, A and B), AtSDR4L direct targets were identified by chromatin immunoprecipitation-sequencing (ChIP-seq) using ABA-treated seedlings 1 d after imbibition (1 DAI) (Supplementary Table S3). Genomic regions enriched with either VAL2 or VAL1 were identified by differential binding (DB) analysis using public data (Yuan et al. 2021) (Fig. 2A, Supplementary Fig. S2C). We showed that both AtSDR4L and DIG1 share extensive binding overlaps with VAL2 and are enriched in regions preferred by VAL2 (Fig. 2A, Supplementary Fig. S2, C and D), supporting a direct interaction with VAL2. AtSDR4L and DIG1 are also found in regions bound by both VALs, suggesting that they might indirectly interact with VAL1 through VAL1–VAL2 heterodimerization (Chen et al. 2020). RY (CATGCA/TGCATG) and G-box (CACGTG) motifs are among the top enriched motifs in the VAL1- and VAL2-preferred regions, respectively (Fig. 2B). The enrichment of G-box in VAL2-preferred regions echoes with the enrichment of this motif in AtSDR4L and DIG1 target sites (Fig. 2, B and C) (Song et al. 2016; Wu et al. 2022). Previously, a G-box-binding TF ABA INSENSITIVE 5 (ABI5) was shown to interact with DIG1 in Y2H (Lumba et al. 2014). Plausibly, DIG1 and AtSDR4L bring VAL2 and G-box-binding TFs into close proximity, enabling a robust and temporal-specific repression of seed maturation genes.

## A key set of transcriptional regulators targeted by AtSDR4L-VAL2 have reduced H3K27me3 in *Atsdr4l* mutants

Because VAL proteins interact with PRC1 and PRC2 components, and that both PRCs affect H3K27me3 directly or indirectly (Yuan et al. 2016; Chen et al. 2020; Baile et al. 2021; Yuan et al. 2021), we examined H3K27me3 in Col-0, *Atsdr4l-4*, and *Atsdr4l-5* seedlings and identified hundreds of DB regions (Fig. 2C, Supplementary Fig. S2, E, G, and H, Supplementary Table S4). Despite more DB regions showing elevated H3K27me3 in *Atsdr4l* mutants, AtSDR4L protein exhibits stronger binding near the sites of reduced H3K27me3 (Supplementary Fig. S2F), suggesting that loci with increased H3K27me3 in *Atsdr4l* mutants are not directly regulated by AtSDR4L. Interestingly, a region upstream of *ABA INSENSITIVE 3* (*ABI3*) has reduced H3K27me3 in *Atsdr4l* at 3 DAI (Fig. 2C). Previously, we reported a peak with low signal and reproducibility by inducible AtSDR4L ChIP near this H3K27me3 DB region (Wu et al. 2022). The binding is significant (*q*-value < 0.05) in both replicates of *AtSDR4Lpro::3xHA-AtSDR4L-3xFLAG* lines, possibly because of strong AtSDR4L expression in 1-DAI ABA-treated seedlings (Supplementary Fig. S2B). This distal upstream region might function along with the RY motifs in the 3′UTR to regulate *ABI3* expression (Liang et al. 2022). Consistently with the inducible AtSDR4L lines, AtSDR4L in the native promoter lines binds to the upstream of *LEAFY COTYLEDON1* (*LEC1*) (Fig. 2C). However, H3K27me3 accumulates to a similar level at 1 DAI in wild-type and *Atsdr4l* seedlings (Fig. 2C), suggesting that the epigenetic regulation of *LEC1* might occur earlier in maturing seeds. Additionally, H3K27me3 is significantly (false discovery rate < 0.05) decreased at *AtSDR4L* and its paralogous loci *DIG2* and *DIL1* in the *Atsdr4l* mutants at 3 DAI (Fig. 2C). AtSDR4L peaks were identified in the native promoter lines upstream of these loci, indicating a feedback mechanism to repress these co-repressor genes in seedling establishment by AtSDR4L-mediated PRC recruitment. AtSDR4L is highly expressed in maturing and dry seeds (Cao et al. 2020; Liu et al. 2020). The expression of *LEC1* and *ABI3* is modestly altered in *val1 val2* and *Atsdr4l* maturing seeds, often by less than 5 to 10-fold (Jia et al. 2013; Zheng et al. 2022). The magnitude of derepression is substantially higher in mutant seedlings (Jia et al. 2013; Wu et al. 2022; Zheng et al. 2022). Therefore, we propose that AtSDR4L and DIG1 mark genes subject to VAL- and PRC-mediated repression in maturing and germinating seeds, prior to the massive deposition and spreading of H3K27me3 during seedling establishment (Fig. 2D).

## Supplementary Material

kiae225_Supplementary_Data

## Supplementary data

The following materials are available in the online version of this article.

**Supplementary Materials and Methods**.

**Supplementary Figure S1**. AtSDR4L, DIG1, and DIL1 physically interact with VAL2.

**Supplementary Figure S2**. AtSDR4L affects the deposition of H3K27me3 during early seedling establishment.

**Supplementary Table S1**. pDEST22 clones for Y2H.

**Supplementary Table S2**. Primers used in this study.

**Supplementary Table S3**. Peak lists of *AtSDR4Lpro*::3xHA-AtSDR4L-3xFLAG ChIP-seq.

**Supplementary Table S4**. H3K27me3 DiffBind regions in *Atsdr4l* mutants at 1 and 3 DAI.

## Dive Curated Terms

The following phenotypic, genotypic, and functional terms are of significance to the work described in this paper:

ABI3 Gramene: AT3G24650
ABI3 Araport: AT3G24650

seed AmiGo: PO:0009010

seed dormancy Planteome: TO:0000253

AtSDR4L Gramene: AT1G27461
AtSDR4L Araport: AT1G27461

ABI5 Gramene: AT2G36270
ABI5 Araport: AT2G36270

h3k27me3 CHEBI: CHEBI:85045

VAL1 Gramene: AT2G30470
VAL1 Araport: AT2G30470

DIL1 Gramene: AT5G63350
DIL1 Araport: AT5G63350

VAL2 Gramene: AT4G32010
VAL2 Araport: AT4G32010

LEC1 Gramene: AT1G21970
LEC1 Araport: AT1G21970

DIG1 Gramene: AT3G48510
DIG1 Araport: AT3G48510

DIG2 Gramene: AT5G50360
DIG2 Araport: AT5G50360
