# Supplementary material for: Co-repressors AtSDR4L and DIG1 interact with transcription factor VAL2 and promote Arabidopsis seed-to-seedling transition
Source: Plant Physiol. 2024 Apr 23;195(4):2528–32. doi: 10.1093/plphys/kiae225 (PMC11288726; doi:10.1093/plphys/kiae225)
Supplement: kiae225_Supplementary_Data [file kiae225_supplementary_data.zip › SupplementaryData.docx]

Supplementary Data

## Co-repressors AtSDR4L and DIG1 interact with transcription factor VAL2 and promote Arabidopsis seed-to-seedling transition

## Supplementary Materials and Methods

**Plant materials and growth conditions**

Arabidopsis (*Arabidopsis thaliana*) seeds were surface sterilized and sown on 1x LS Linsmaier & Skoog (LS) medium (LSP03, Caisson Labs, Smithfield, UT, USA) supplemented with 1% (w/v) sucrose and 0.8% (w/v) plant agar. Seeds were stratified for 3 days at 4 °C, and then grown on plates for 7 to 10 days before being transferred to soil to grow under 8 h dark / 16 h light conditions at 22 °C. Both *Atsdr4l-4* and *Atsdr4l-5* carry segmental deletions in the Col-0 background. *Atsdr4l-4* contains a 101-bp deletion, and was segregated from Allele 1 of a previously reported biallelic mutant (Wu et al., 2022). To generate *Atsdr4l-5*, two guide RNA spacers were introduced to pHEE401E using primers listed in Table S2 following a published protocol (Wang et al., 2015). The resulting pHEE401E-AtSDR4Lsg was introduced into the *Agrobacterium tumefaciens* strain GV3101 before the transformation of wild-type Col-0 using the floral dip method (Clough and Bent, 1998). T1 plants of *pHEE401E-AtSDR4Lsg* were selected on LS medium supplemented with 25 µg/mL Hygromycin B (H-270-1, GoldBio, USA). A mutant line carrying a 80-bp deletion was identified and backcrossed with wild-type Col-0. The zCas9-free BC1F2 lines of *Atsdr4l-5* were bulked for physiological characterization and histone ChIP. The physiological characterization was performed for 3 biological replicates as previously described (Wu et al., 2022). In brief, *Atsdr4l-5* and Col-0 seeds were after-ripened for 10 weeks. The seeds were then either stratified at 4 °C in dark for 3 days or directly sowed on 1X LS medium supplemented either with or without 1% (w/v) sucrose. Six-day-old seedlings were scored for embryonic traits.

To generate the *AtSDR4Lpro::3xHA-AtSDR4L-3xFLAG* lines, a 1270-bp *AtSDR4L* native promoter, *AtSDR4L* coding sequence with an N-terminal 3xHA tag and a C-terminal 3xFLAG tag, and a pCAMBIA1300 vector linearized by EcoRI and PstI were assembled using the NEBuilder® HiFi DNA Assembly Master Mix following the manufacturer’s instructions (E2621S, NEB, Ipswich, MA, USA). The resulting pCAMBIA1300-AtSDR4Lpro::3xHA-AtSDR4L-3xFLAG was transformed into NEB 10-beta Chemically Competent *Escherichia. coli* (C3019H, NEB, Ipswich, MA, USA), and then introduced into the *A. tumefaciens* strain C58 before the transformation of *Atsdr4l-4* by floral dip. Transgenic lines were selected on LS medium supplemented with 25 µg/mL Hygromycin B. The lines carrying a single-positioned insertion were identified by Chi-squared test, and homozygous lines at the T4 generation were used for western blotting and ChIP-seq. Primers used for the cloning and validation of *Atsdr4l* mutants and the *AtSDR4Lpro::3xHA-AtSDR4L-3xFLAG* transgenic lines are listed in Table S2.

**Yeast two-hybrid assay**

Coding sequences for DIG1 and AtSDR4L were cloned into a pENTR/D-TOPO vector and subsequently transferred into the pDEST32 vector using Gateway™ LR Clonase™ II Enzyme mix according to the manufacturer’s instructions (11791020, Thermo Fisher Scientific, Waltham, MA, USA). A list of candidate prey genes consisting of PRC core and accessory proteins was generated, and the corresponding pDEST22 or pDEST-AD clones were ordered from the ABRC (Table S1). Protein structures of DIG1 and AtSDR4L were predicted by AlphaFold (Jumper et al., 2021) and later by AlphaFold2 using the MMseqs2 algorithm (ColabFold v1.5.5) (Mirdita et al., 2022). The program was run under the following default parameters: msa_mode of "mmseqs2_uniref_env" and pair_mode of "unpaired_paired". Resulting top-ranked protein structures were visualized using PyMOL™ 2.5.7. All plasmids for the truncations of AtSDR4L, DIG1, and VAL2 were generated by the amplification of the desired fragments using primers listed in Table S2, and the subsequent ligation into the pDEST22 or pDEST32 vector through the NotI and AscI sites. Bait and prey plasmids were transformed into yeast (*Saccharomyces cerevisiae)* Y2HGold competent cells. Transformants were selected on double drop-out (DDO) media lacking Leu and Trp (-LW). Independent colonies were suspended in liquid DDO media and incubated overnight at 30°C. The cultures were then centrifuged and resuspended in sterile water, adjusting to an OD600 of 1.0. For the selection plates, 5 µL of undiluted, 1:10 (v/v) dilution, and 1:100 (v/v) dilution were used. When testing for the protein-protein interaction, the transformed yeast was grown on triple drop-out (TDO) media lacking Leu, Trp, and His (-LWH). 3-amino-1,2,4-triazole (3-AT) was added to the TDO media at a gradient of concentrations such as 0 mM, 0.1 mM, and 1 mM. Yeast plated on DDO and TDO plates were scanned after 2 days and 4 days of incubation, respectively.

**Bimolecular fluorescence complementation (BiFC) assay**

Full-length or truncated VAL2 coding sequence without a stop codon and full-length DIG1 coding sequence with a stop codon were PCR amplified using the primers listed in Table S2 and cloned into a NotI- and AscII-linearized pENTR™/D-TOPO™ plasmid (K240020, Thermo Fisher Scientific, Waltham, MA, USA) using the NEBuilder® HiFi DNA Assembly Master Mix (E2621S, NEB, Ipswich, MA, USA) to generate the entry clones, which were subsequently recombined using the Gateway™ LR Clonase™ II Enzyme mix (11791020, Thermo Fisher Scientific, Waltham, MA, USA) into the destination vectors to make *pUB-VAL2-nYFP-Dest*, *pUB-nVAL2-nYFP-Dest*, and *pUB-cYFP-DIG1-Dest* that drive the expression of the fusion proteins with the UBQ10 promoter. *Nicotiana benthamiana* seeds were stratified for 3 days at 4 °C, sown in soil, and grown under long-day (8-h dark / 16-h light) conditions at 22 °C for more than 5 weeks before injection. For BiFC, *A. tumefaciens* strain GV3101was transformed with the pUB plasmids, incubated overnight at 30 ºC with gentle shaking at 200 rpm in 4 mL LB medium containing gentamicin (25 µg / mL), rifampin (25 µg / mL), and kanamycin (50 µg / mL). Cells were pelleted at 4,000 rpm for 6 minutes at room temperature. After the removal of supernatant, the cells were gently resuspended in 1 mL of 10 mM MgCl_2_ and centrifuged in a 1.5 mL microcentrifuge tube at 5,000 rpm at room temperature for 4 minutes. After repeating the centrifugation and resuspension steps, 100 μL of each resuspended sample was diluted to an O.D.600 value 0.4. Diluted *A. tumefaciens* containing the nYFP and cYFP plasmids were mixed at a 1:1 (v/v) ratio, and gently infiltrated using 1 mL syringes without a needle into the underside of *N. benthamiana* leaves. The injected leaves were incubated at 22 °C in darkness for 2 days. Yellow fluorescent protein signal was captured utilizing the Olympus FV1000 multiphoton confocal laser-scanning microscope with Olympus Fluoview software (FV10-ASW2) at the UBC Bioimaging Facility. The images were acquired using a 30X UPLSAPO (1.05 N.A.) objective lens, a 514-nm laser for YFP excitation, and an emission range of 530 nm – 630 nm for signal capture. Imaging was carried out under “Analog” mode of photomultiplier tube (PMT) with a PMT gain at 1.0 and offset at 6. Raw images were further cropped by ImageJ 2.1.0 (Schneider et al., 2012).

**RT-qPCR**

Wild-type Col-0 seeds were surface sterilized by 50% (v/v) bleach and cold stratified at 4 °C for 3 days, and then plated on 1X LS medium for 38, and 70 hours. Germinating seeds from each time point was incubated in 5 uM abscisic acid (ABA) or mock solution for 5 hours on agar plates before harvest. Total RNA was extracted from Col-0 dry seeds and ABA- or mock-treated seedlings using the Spectrum™ Plant Total RNA Kit (STRN50, MilliporeSigma, Burlington, MA, USA). DNase I treatment was carried out on RNA samples to remove remnant genomic DNA (AM1907, TURBO DNA-free™ Kit, Thermo Fisher Scientific, Waltham, MA, USA). Subsequently, cDNA was synthesized using Maxima H Minus Reverse Transcriptase (Thermo Fisher Scientific, Waltham, MA, USA, EP0752). PCR was performed using the PowerUp™ SYBR™ Green Master Mix (Thermo Fisher Scientific, Waltham, MA, USA, A25742) in a QuantStudio™ 3 System (Thermo Fisher Scientific, Waltham, MA, USA) with 3 biological replicates. Fold change was calculated using the ΔΔCT approach using *ACTIN8* as the endogenous control for normalization. Error bars are computed as mean value +/- standard error of the mean (SEM) from 3 biological replicates.

**Western blotting**

For the Arabidopsis samples, *AtSDR4Lpro::3xHA-AtSDR4L-3xFLAG* and *Atsdr4l-4* seeds were surface sterilized by 50% (v/v) bleach and cold stratified at 4 °C for 3 days, and then plated on 1X LS medium for 38, 46, 70, and 94 hours. Germinating seeds from each time point were incubated in 5 uM ABA or mock solution for 5 hours on the agar plates before harvest. Proteins were extracted from *AtSDR4Lpro::3xHA-AtSDR4L-3xFLAG* transgenic samples and *Atsdr4l-4* mutants in 2X LDS buffer (NP0008, Thermo Fisher Scientific, Waltham, MA, USA) with 10 mM Dithiothreitol (DTT10, Gold Biotechnology, St Louis, MO, USA) and 1% (v/v) protease inhibitor (P9599, MilliporeSigma, Burlington, Massachusetts, USA).

For the *N. benthamiana* samples, *A. tumefaciens* hosting binary vectors of *pCAMBIA1300-35Spro-3HA-DIG1*, *pCAMBIA1300-35Spro-3HA-AtSDR4L*, *pCAMBIA1300-35Spro-VAL2-3FLAG*, *pCAMBIA1300-35Spro-nVAL2-3FLAG*, and *pCAMBIA1300-35Spro-eYFP-NLS-3FLAG* (generated using primers listed in Table S2) were injected into 2 replicates of the *N. benthamiana* leaves in the presence or absence of RNA silencing suppressor p19 following the same procedure as described in the BiFC assay. Approximately 30 mg of *N. benthamiana* leaves were collected and placed in a 1.5 mL microcentrifuge tube along with a metal bead. The tubes were frozen immediately by liquid nitrogen, and loaded onto adaptors that were prechilled at -20 °C. Subsequently, the samples were homogenized using a TissueLyser (Qiagen, Hilden, Germany) at the oscillation frequency of 30 Hz for 30 seconds. Homogenized powder was rapidly spinned at 4 °C, and mixed by vortexing with 80 μL of the lysis buffer consisting of 2x NuPAGE™ LDS sample buffer (NP0008, Thermo Fisher Scientific, Waltham, MA, USA) and 3% β-Mercaptoethanol (MilliporeSigma, Burlington, Massachusetts, USA). The lysate was incubated at 70 °C for 10 minutes, spinned at maximum speed for 2-3 minutes, and the supernatant was used for western blotting.

Lysed samples were subsequently resolved in 4 to 12% Bis‐Tris gel (NP0322, Thermo Fisher Scientific, Waltham, MA, USA) and transferred to a polyvinylidene fluoride membrane (162-0177, BioRad, Hercules, CA, USA). The membrane was incubated with the primary anti‐HA antibody (C29F4, 1:1000 v/v dilution, CST, Danvers, MA, USA), anti-FLAG (1804, 1:2000 v/v dilution, MilliporeSigma, Burlington, Massachusetts, USA), or anti‐H3 antibody (ab1791, 1:10000 v/v dilution, Abcam, Cambridge, UK), at 4 °C overnight. Incubation with the secondary antibody was performed at room temperature for 1 hour using horseradish peroxidase‐linked anti‐rabbit IgG (7074, 1:10000 v/v dilution, CST, Danvers, MA, USA) or anti-mouse IgG (7076, 1:10000 v/v dilution, CST, Danvers, MA, USA). Clarity Max Western ECL Substrate (1705062, BioRad, Hercules, CA, USA) or SuperSignal™ West Atto Ultimate Sensitivity Substrate (A38555, Thermo Fisher Scientific, Waltham, MA, USA) was incubated with the membrane for 5 minutes at room temperature for visualization under 425 nm wavelength in a ChemiDoc™ Touch Imaging System (BioRad, Hercules, CA, USA).

**Chromatin immunoprecipitation sequencing**

ChIP procedure was performed according to a published protocol and previously reported procedure for the estradiol-inducible AtSDR4L lines (Song et al., 2016b; Wu et al., 2022). For 3xHA-AtSDR4L-3xFLAG ChIP, 1-2 grams of *AtSDR4Lpro::3xHA-AtSDR4L-3xFLAG* transgenic (from 2 replicates) and wild-type germinating seeds were harvested at 1 day after imbibition (DAI) on 1× LS medium supplemented with 5 μM ABA. ABA was added 5 hours prior to sample collection. For H3K27me3 profiling, approximately 0.3 grams of *Atsdr4l* and wild-type samples (each with 2 replicates) were harvested at 1 DAI and 3 DAI which were grown on 1× LS medium supplemented with 1% (w/v) sucrose. After formaldehyde cross-linking and nuclei isolation, nuclei were lysed and sonicated for 15 cycles of 30‐s ON and 90-s OFF at HIGH setting in a Bioruptor Plus (Diagenode, Denville, NJ, USA). Sonicated chromatin was diluted by ChIP Dilution Buffer (Song et al., 2016b) and then incubated overnight with HA antibody (3724, 1:100 v/v dilution, CST, Danvers, MA, USA) and H3K27me3 antibody (61018, 1:100 v/v dilution, Active Motif, Carlsbad, CA, USA) for the precipitation of 3xHA-AtSDR4L-3xFLAG and H3K27me3 bound chromatin fragments, respectively. These antibodies were bound to Dynabeads protein A and G (10001D and 10003D, Thermo Fisher Scientific, Waltham, MA, USA) before capturing chromatin. DNA was reverse crosslinked at 65°C for 6 h, followed by treatment of 100 μg/mL proteinase K at 55°C for 2 h before the phenol:chloroform:IAA (25:24:1 v/v, pH 8.0) extraction. DNA was precipitated and double‐size selected using 0.6 vol. and 1.3 vol. AMPure XP beads (A63881, Beckman Coulter, Brea, CA, USA). Subsequently, libraries were constructed using the NEBNext® Ultra™ II DNA Library Prep Kit for Illumina® (E7645S, NEB, Ipswich, MA, USA) and sequenced on Illumina NovaSeq 6000.

**Data analyses**

For the ChIP-seq libraries of AtSDR4L and H3K27me3, reads were analyzed by MultiQC v1.11. Low-quality reads were filtered, and adapters were trimmed by fastp version 0.23.4 (Chen et al., 2018). Reads were aligned to the Arabidopsis genome (TAIR10) (Lamesch et al., 2012) by Bowtie 2 v2.2.5 with the default parameters (Langmead and Salzberg, 2012). Subsequently, the aligned reads were converted to binary alignment (BAM) format and filtered for mapping quality scores greater than 10 by Samtools v1.6 (Li et al., 2009). Alignment files of mock IgG controls from wild-type and mutant backgrounds were concatenated to form a common control for peak calling of H3K27me3 libraries. For H3K27me3 ChIP-seq, peaks were called by MACS2 v2.2.7.1 using the combined mock IP as a control (Zhang et al., 2008). The argument “--broad” was specified for H3K27me3 libraries to call broad peaks. The arguments “-f BAMPE --keep-dup auto -g 1.2e8 -q 0.1” were used to specify pair-end library, maximum tag calculation, effective genome size of 1.2 Mb, and q-value cut-off at 0.1 for all libraries. The remaining arguments were the same as the default MACS2 settings (Zhang et al., 2008). Quality assessment report of the AtSDR4L native promoter ChIP-seq was generated using R package ChIPQC_1.30.0 (R Version: 4.1.2) (Carroll et al., 2014; The R Development Core Team, 2020).

ChIP-seq data for estradiol-inducible AtSDR4L (4-day old seedlings) was obtained from GSE185388 (Wu et al., 2022), VAL1 and VAL2 data (14-day old seedlings) were downloaded from PRJNA607059 (Yuan et al., 2021), and DIG1 and DIG2 data (4-day old seedlings) were obtained from PRJNA319317 (Song et al., 2016a). Adapter trimming for VAL1 and VAL2 libraries was performed using fastp v0.12.4 (Chen et al., 2018). For all public ChIP-seq libraries, reads were mapped to the TAIR10 reference genome (Lamesch et al., 2012) using Bowtie2 v2.3.5.1 with the default parameters (Langmead and Salzberg, 2012). The alignments were converted to BAM and filtered for mapping quality scores greater than 10 on Samtools v1.11 (Li et al., 2009). Peak calling for published TF ChIP and native promoter-driven AtSDR4L ChIP libraries was performed on MACS2 v2.2.6 (Zhang et al., 2008) using the uninduced sample (estradiol-inducible AtSDR4L), Col-0 wild-type (native promoter-driven AtSDR4L), GFP construct without DIG fusion (DIG1/DIG2), or input samples (VAL1/VAL2) as controls. For AtSDR4L, VAL1 and VAL2 ChIP-seq, the argument “-f BAMPE” was used to specify the pair-end library, and “-g 1.2e8 -q 0.1 --call-summits” were specified for all libraries to indicate effective genome size of 1.2 Mb, q-value cut-off at 0.1, and calling of subpeaks. The default MACS2 settings were applied to the remaining arguments (Zhang et al., 2008). NarrowPeak files were generated as the outputs by MACS2, which were then sorted by q-values and the reproducible peaks were merged (for 2 replicates from AtSDR4L and VAL1/VAL2) using IDR version 2.0.4.2 by q-value rank and “min” merge method (Li et al., 2011). The tiled data files (TDF) were created from BAM files by igvtools v2.5.3, with the maximum zoom level of 5 for precomputing, window size of 10 bp for averaging coverage, and “includeDuplicates” option enabled (Robinson et al., 2011). Visualization of ChIP-seq tracks were generated on Integrative Genomics Viewer 2.9.4 (Robinson et al., 2011).

NarrowPeak format data were imported into RStudio version 2021.09.2+382 for filtering and coordinate sorting (RStudio Team, 2020; The R Development Core Team, 2020). To consider significance of TF binding, reproducible peaks from AtSDR4L, VAL1 and VAL2 ChIP-seq were filtered by Irreproducible Discovery Rate (IDR) ≤ 0.1 (score ≥ 415), and DIG1 and DIG2 peaks (no replicates) were filtered by q-value < 0.05 using tidyverse version 1.3.2 (Wickham et al., 2019). The venn diagram for overlaps between different TF binding sites were generated using “findOverlapsOfPeaks” and “makeVennDiagram” functions in ChIPpeakAnno package v1.3.1000, and all IDR-merged peaks from AtSDR4L, VAL2 and VAL1 were included. NarrowPeak and BAM files from individual VAL1 and VAL2 ChIP replicates were processed by DiffBind package v3.4.11 in R, and the contrasts were built between the two proteins to perform differential enrichment/binding (DB) analysis for genomic regions +/- 200 bp from the peak summits (Rory and Brown, 2011). Genomic regions that are significantly differentially enriched with false discovery rate (FDR) < 0.05 for either protein were determined by DEseq2 and extracted separately, and the non-DB groups were kept using tidyverse v1.3.2 (Love et al., 2014). Regions corresponding to organellar genomes were removed. Genomic coordinates for VAL1 > VAL2, VAL2 > VAL1, and non-DB regions were saved as BED files. DNA sequences of +/- 100 bp from all TFs’ significant peak summits, as well as 401-bp significant DB regions from VAL1-VAL2 comparison were obtained from BSgenome.Athaliana.TAIR.TAIR9 1.3.1000 (Pagès, 2021). Summit sequences that overlap with highly repetitive elements documented in RepetDB were removed before motif discovery (Amselem et al., 2019). Motif enrichment was carried out on MEME Suite (Bailey et al., 2015). Summit sequences extracted from previous steps were input to MEME-ChIP program v5.5.3 with the shuffled input sequences as the control (Machanick and Bailey, 2011). Motifs with E-values < 0.05 were considered enriched.

Differential enrichment/binding (DB) of H3K27me3 between wild-type and *Atsdr4l-4* or *Atsdr4l-5* at 1 DAI and 3 DAI were computed on RStudio v2023.06.01 (RStudio Team, 2020; The R Development Core Team, 2020) using the same DiffBind method (package v3.10.0) as described for VAL1-VAL2 comparison above (Rory & Brown, 2011), except that +/- 400 bp was defined for the summit setting to compute H3K27me3 DB. Regions with fold > 0 and FDR < 0.1 were considered significantly reduced (Col-0 > *Atsdr4l*) for H3K27me3 signals in *Atsdr4l*, and regions with elevated (*Atsdr4l* > Col-0) signals were filtered by fold < 0 and FDR < 0.1. The filtered sites from *Atsdr4l-4* and *Atsdr4l-5* comparisons were unionized to create the BED files for downstream visualization. Heatmaps and occupancy curves were graphed using deepTools v3.5.1 (Ramírez et al., 2016). BigWig files were created from BAM alignment files of AtSDR4L, DIG1/DIG2, VAL1/VAL2 and H3K27me3 over their respective controls using deepTools function bamCompare, with “BPM” normalization method and “centerReads” option (Ramírez et al., 2016). Score matrix of the bigWig files over the regions defined in the VAL1-VAL2 DiffBind comparison, as well as the H3K27me3 DB regions from the Col-0-*Atsdr4l* comparison, were computed using computeMatrix function, and 3 kb up- and downstream of the region body was specified (Ramírez et al., 2016). The matrix files were then used to create plots for the overall occupancies and heatmaps over the VAL1/VAL2 and H3K27me3 DB regions by “plotHeatmap” function (Ramírez et al., 2016).

**Accession numbers**

AT1G27461 (AtSDR4L), AT3G48510 (DIG1), AT4G32010 (VAL2), AT2G30470 (VAL1), AT1G21970 (LEC1), AT3G24650 (ABI3), AT5G50360 (DIG2), AT5G63350 (DIL1), AT5G40790 (DIL2), AT5G40800 (DIL3), AT3G27250 (DIL4), AT1G49240 (ACTIN8), Os07g0585700 (rice Sdr4).

## Data Availability

ChIP-seq data in the article were submitted to the NCBI Gene Expression Omnibus with accession number: [GSE246997](https://www.ncbi.nlm.nih.gov/geo/query/acc.cgi?acc=GSE246997).

###

###


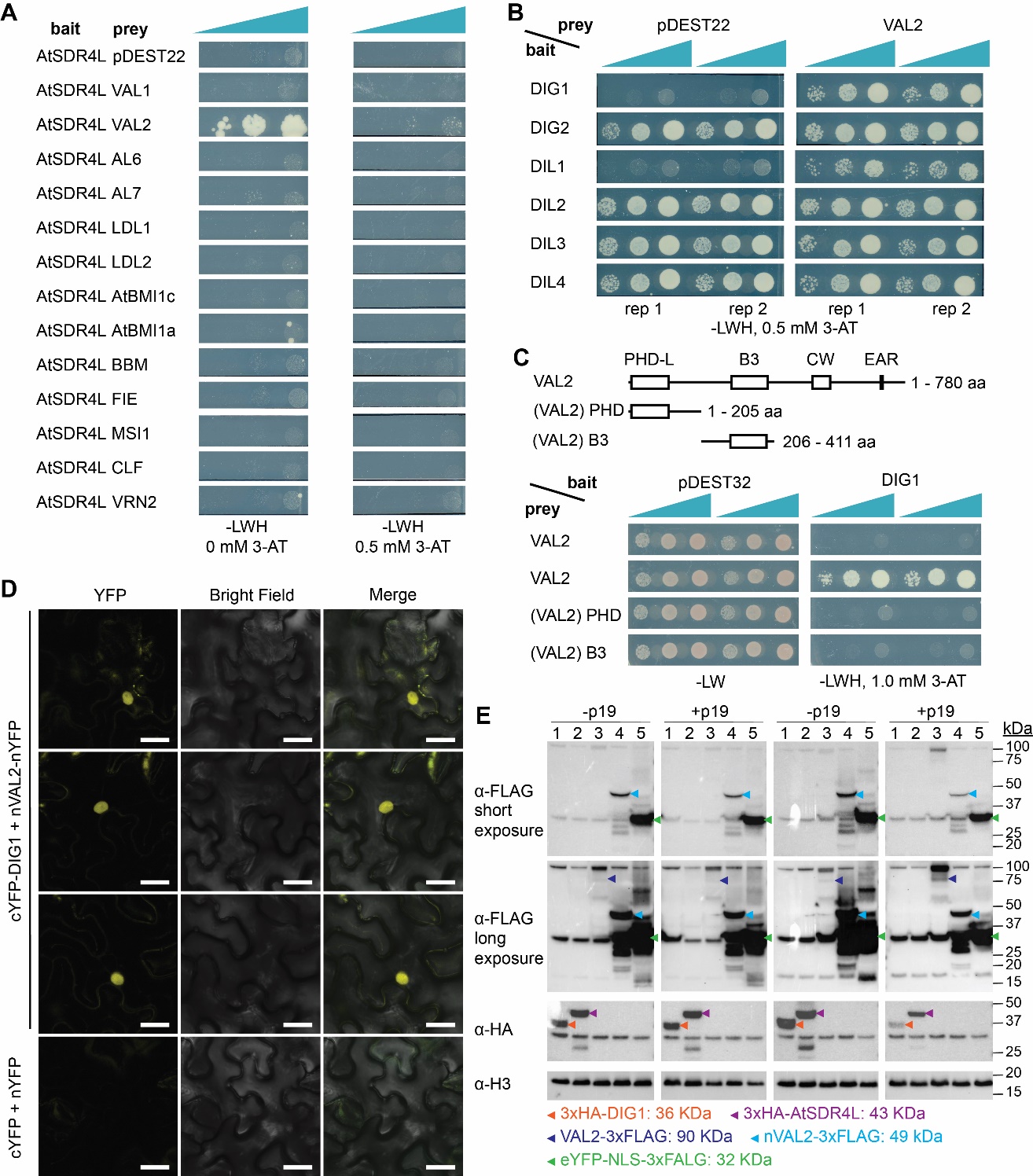


Supplementary Figure S1. AtSDR4L, DIG1, and DIL1 physically interact with VAL2. **A)** Yeast-two-hybrid assays using yeasts co-expressing the indicated pairs of bait and prey show that AtSDR4L physically interacts with VAL2 but not with other histone modification proteins. Bait proteins were expressed from the pDEST32 vector that encodes a GAL4 DNA binding domain and prey proteins were expressed from the pDEST22 vector that encodes a GAL4 DNA activation domain. Yeasts were plated on triple dropout media supplemented with different concentrations of 3-amino-1,2,4-triazole (3-AT) on a dilution gradient of 1:100 (v/v), 1:10 (v/v), and undiluted (shown by triangles above each lane). -L, -W, -H: dropout media deprived of leucine, tryptophan, and histidine. **B)** Yeast-two-hybrid assay shows that DIG1 and DIL1 physically interact with VAL2. DIG2, DIL2, DIL3, and DIL4 exhibit strong autoactivation, and their physical interaction with VAL2 cannot be determined. **C)** Yeast-two-hybrid assay shows that neither the PHD-L (plant homeodomain-like) nor B3 domain of VAL2 is sufficient to interact with DIG1. **D)** Bimolecular fluorescence complementation assay showing the interaction between DIG1 and the N-terminal half of VAL2 in three nuclei from a single *Nicotiana benthamiana* leaf. Positive interactions are illustrated by the YFP signal. Scale bar = 20 μm. **E)** Western blot results for transient expression of 3xHA-DIG1 (lane 1), 3xHA-AtSDR4L (lane 2), full-length (lane 3) and N-terminal half (lane 4) of VAL2 tagged with 3xFLAG, and eYFP with 3xFLAG tag and nuclear localization signal (NLS) (lane 5) in *N. benthamiana* leaves, in the presence and absence of RNA silencing suppressor p19. The left and right halves of the panel were generated from two individual replicates. Arrowheads indicate the predicted sizes for each protein and are color-coded with the construct labels accordingly. Histone 3 (~17 kDa) was used as the loading control.


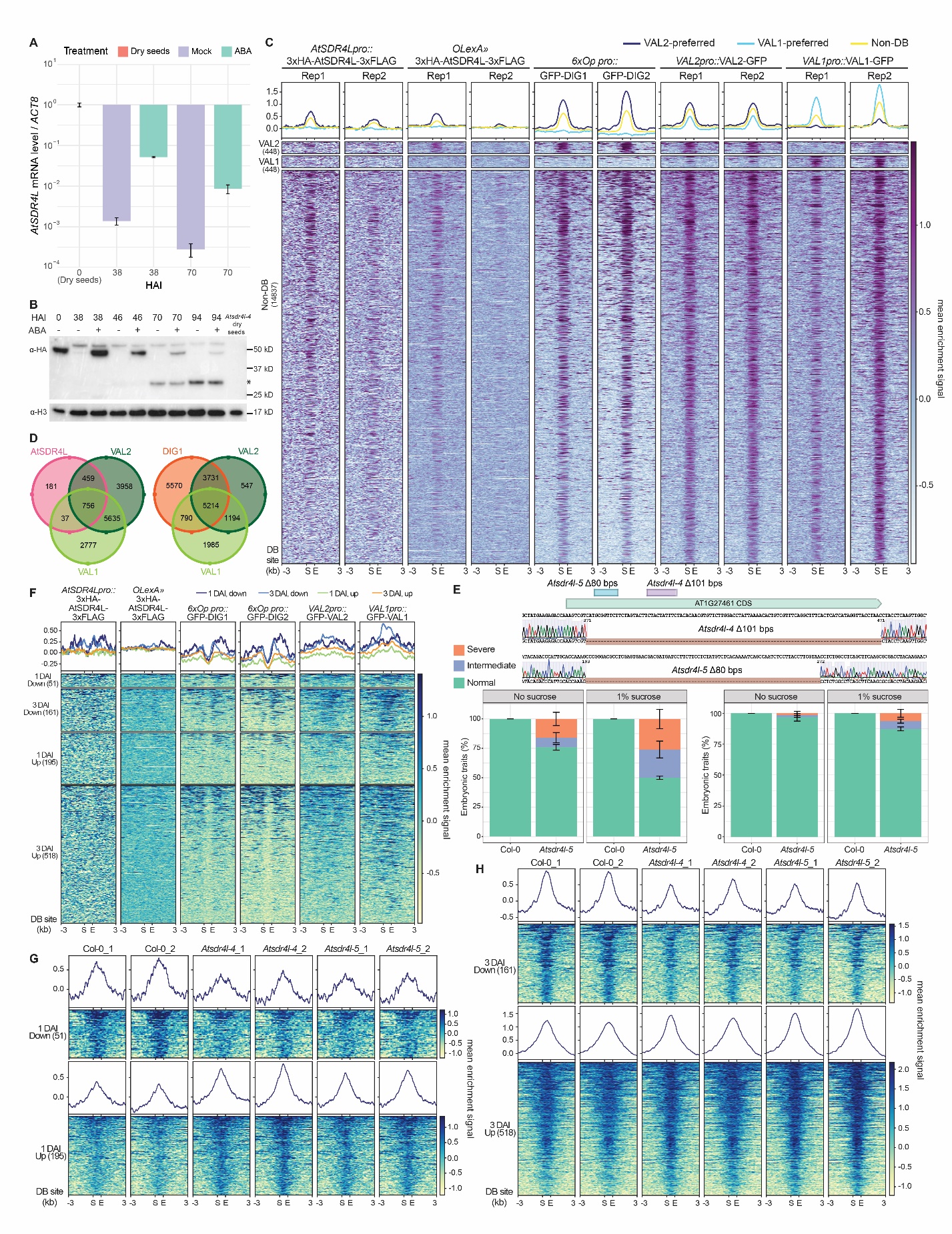


Supplementary Figure S2. AtSDR4L affects the deposition of H3K27me3 during early seedling establishment. **A)** *AtSDR4L* mRNA accumulation during seedling establishment. *AtSDR4L* transcript abundance was normalized to *ACTIN8* and presented relative to its level in the dry seeds. Seedling samples were subjected to 5 hours of 5-µM abscisic acid (ABA) or mock treatment prior to harvest at the indicated hours after imbibition (HAI). Error bar represents the mean value +/- SEM using 3 biological replicates. **B)** Expression of 3xHA-AtSDR4L-3xFLAG (~46.5 kDa) at indicated HAI after 5 hours of 5-µM ABA or mock treatment. Dry seeds of *Atsdr4l-4* were included as the negative control. An asterisk at ~ 30 kDa indicates non-specific probing by the anti-HA antibody in seedlings older than 2 days after imbibition (DAI). Histone 3 (~17 kDa) was used as the loading control. **C)** Profile plots and heatmaps representing the enrichment signal (mean normalized log2 ratio relative to respective control) of AtSDR4L, DIG1, DIG2, VAL2 and VAL1 over the regions preferred by VAL2 and VAL1, as well as all the non-differentially bound (non-DB) regions from VAL1-VAL2 DiffBind analysis. S = peak start, E = peak end. **D)** Venn diagrams of ChIP-seq peaks overlap among AtSDR4L (native promoter lines), VAL2 and VAL1 (left), and among DIG1, VAL2 and VAL1 (right). **E)** Sequence alignments and coding sequence (CDS) map for the positions of deletions in *Atsdr4l-4* and *Atsdr4l-5* mutants. Proportions of the embryonic phenotypes in non-stratified (left) and 3-day (4°C) stratified (right) *Atsdr4l-5* mutants and wild-type Col-0 seedlings at 6 DAI in the absence and presence of 1% (w/v) sucrose. Seeds were after-ripened for 10 weeks before assaying. Error bar represents the mean value +/- SEM using 3 biological replicates. **F)** Profile plots and heatmaps for the enrichment signal (mean normalized log2 ratio relative to respective control) of AtSDR4L, DIG1, DIG2, VAL2 and VAL1 in regions with decreased (down) and increased (up) H3K27me3 signals unionized from two *Atsdr4l* mutants at 1 DAI and 3 DAI. **G-H)** Profile plots and heatmaps for the enrichment signal (mean normalized log2 ratio relative to combined mock control) of H3K27me3 in Col-0, *Atsdr4l-4* and *Atsdr4l-5* samples, over the regions with decreased (down) and increased (up) H3K27me3 signals unionized from both *Atsdr4l* mutants at 1 DAI and 3 DAI.

###

### Supplementary Tables

Supplementary Table S1: pDEST22 clones for Y2H

Supplementary Table S2: primers used in this study

Supplementary Table S3: Peak lists of *AtSDR4Lpro*::3xHA-AtSDR4L-3xFLAG ChIP-seq (separate spreadsheet)

Supplementary Table S4: H3K27me3 DiffBind regions in *Atsdr4l* mutants at 1 and 3 DAI (separate spreadsheet)

**Supplementary Table S1: pDEST22 clones for Y2H**

| **Prey name** | **AGI locus identifier** | **ABRC clone** |
| --- | --- | --- |
| VAL1 | AT2G30470 | DEST-U21-A01 |
| VAL2 | AT4G32010 | DEST-U19-A01 |
| AL6 | AT2G02470 | DEST-U13-F01 |
| AL7 | AT1G14510 | DEST-U20-A10 |
| LDL1 | AT1G62830 | DEST-U19-D03 |
| LDL2 | AT3G13682 | DEST-U20-G05 |
| AtBMI1c | AT3G23060 | pDEST-AD048C04 |
| AtBMI1a | AT2G30580 | DEST-U03-G12 |
| BBM | AT5G17430 | DEST-U15-C07 |
| FIE | AT3G20740 | pDEST-AD042C04 |
| MSI1 | AT5G58230 | pDEST-AD097D03 |
| CLF | AT2G23380 | DEST-U20-H03 |
| VRN2 | AT4G16845 | DEST-U12-H03 |

**Supplementary Table S2: primers used in this study**

**Cloning primers - Y2H and Arabidopsis transformation**

| **Construct** | **primer name** | **primer sequence (5’ - 3’)** |
| --- | --- | --- |
| pDEST22-VAL2-N | NotI-start-VAL2_F | aaGCGGCCGCccccttcaccATGGAGTCAATAAAGGTT |
|  | AscI-VAL2(1-411)-stop_R | aaGGCGCGCCcacccttTCAATTCAAGCTGTTGGAA |
| pDEST22-VAL2-C | NotI-start-VAL2(412-780)_F | aaGCGGCCGCccccttcaccATGCCGGGATGTGGTG |
|  | AscI-VAL2-stop_R | aaGGCGCGCCcacccttTTAGTTCACAGGATCATGA |
| pDEST22-VAL2-PHD | NotI-start-VAL2_F | aaGCGGCCGCccccttcaccATGGAGTCAATAAAGGTT |
|  | AscI-VAL2(1-205)-stop_R | aaGGCGCGCCcacccttTCAAATCGAATTAGTCTTAC |
| pDEST22-VAL2-B3 | NotI-start-VAL2(206-411)_F | aaGCGGCCGCccccttcaccATGTTCCAACTGGCCC |
|  | AscI-VAL2-stop_R | aaGGCGCGCCcacccttTTAGTTCACAGGATCATGA |
| pDEST32-DIG1-C | NotI-DIG1-C_F | aaGCGGCCGCccccttcaccGCTTATCAGACACCGG |
|  | AscI-DIG1-stop_R | aaGGCGCGCCcacccttTCAAAGGCACAAAGCG |
| pDEST32-DIG1-C-Δ8aa | NotI-DIG1-C_F | aaGCGGCCGCccccttcaccGCTTATCAGACACCGG |
|  | AscI-DIG1-8aaTrunc_R | aaGGCGCGCCcacccttTCAGAGCCTCCAAGCAAAAC |
| pDEST32-AtSDR4L-C | NotI-AtSDR4L-C_F | aaGCGGCCGCccccttcacc agcagagtcatatcaccg |
|  | AscI-AtSDR4L-stop_R | aaGGCGCGCCcacccttCTA TCTGCGGTCAGTG |
| pDEST32-AtSDR4L-C-Δ6aa | NotI-AtSDR4L-C_F | aaGCGGCCGCccccttcacc agcagagtcatatcaccg |
|  | AscI-SDR4-6aaTrunc_R | aaGGCGCGCCcacccttTCA gtgaaacctccacgtaaag |
| pHEE401E-Atsdr4l-sg | AtSDR4L_sg_L_F | ATATATGGTCTCGATTGACCCATTGCACCAAAGCCCGTTTTAGAGCTAGAAATAGC |
|  | AtSDR4L_sg_L_R | ATTATTGGTCTCGAAACGAGGTTACGAAGGTAAGGAGAATCTCTTAGTCGACTCTAC |
| pCAMBIA1300-AtSDR4Lpro-3xHA-AtSDR4L-3xFLAG | HiFi_pCAMBIA1300_EcoRI_SDR4pro_F | CAGCTATGACCATGATTACGAATTCCAGAGGATGAATCGGCC |
|  | HiFi_SDR4pro_3xHA_R | catacgggtacatggctagaGACGTCAAGAATGATaaaaaaaaaaaaCTC |
|  | HiFi_SDR4pro_3xHA_F | tttttATCATTCTTGACGTCtctagccatgtacccgtatg |
|  | HiFi_pCAMBIA1300_PstI_3xFLAG_R | GATGATACGAACGAAAGCT CTGCAG TCACTTATCGTCATCGTCCTTG |

**Cloning primers - *Nicotiana benthamiana* infiltration for BiFC and western blotting**

| **Construct** | **primer name** | **primer sequence (5’ - 3’)** |
| --- | --- | --- |
| pUB-VAL2-nYFP-Dest | HiFi_pENTR_NotI_VAL2_F | caaaaaagcaggctccgcggccGCATGGAGTCAATAAAGGTTTGCATGAACGC |
|  | HiFi_pENER_VAL2_AscI_R | aagaaagctgggtcggcgcgCCcGTTCACAGGATCATGAGCTCC |
| pUB-nVAL2-nYFP-Dest | HiFi_pENTR_NotI_VAL2_F | caaaaaagcaggctccgcggccGCATGGAGTCAATAAAGGTTTGCATGAACGC |
|  | HiFi_pENTR_VAL2-N_AscI_R | AAGAAAGCTGGGTC GGCGCGCC C ATTCAAGCTGTTGGAAAACATGTTCAG |
| pUB-cYFP-DIG1-Dest | HiFi_pENTR_NotI_DIG1_F | caaaaaagcaggctccgcggccGCgATGGACGGTAGGGGAGGGTGTTGCATAG |
|  | HiFi_pENER_DIG1_AscI_R | aagaaagctgggtcggcgcgCCTTAAAGGCACAAAGCGGCCTTAACG |
| pCAMBIA1300-35Spro-3xHA-DIG1 | pCambia1300_3xHA_HiFi_F | ggacgagctcggtaccATGTACCCGTATGATGTTCCG |
|  | 3xHA_DIG1_HiFi_R | ccctaccgtcTCCTGCGTAGTCTGGGAC |
|  | 3xHA_DIG1_HiFi_F | ctacgcaggaGACGGTAGGGGAGGGTGTTG |
|  | DIG1_pCambia1300_HiFi_R | atacgaacgaaagctctgcagTTAAAGGCACAAAGCGGCC |
| pCAMBIA1300-35Spro-3xHA-AtSDR4L | pCAMBIA-3xHA-DIG1 | ggacgagctcggtaccATGTACCCGTATGATGTTCCG |
|  | 3xHA_SDR4_HiFi_R | ggttgagtatctttatTCCTGCGTAGTCTGGGAC |
|  | 3xHA_SDR4_HiFi_F | ctacgcaggaATAAAGATACTCAACCCCCAC |
|  | SDR4_pCambia1300_HiFi_R | atacgaacgaaagctctgcagTTATCTGCGGTCAGTGGTTG |
| pCAMBIA1300-35Spro-VAL2-3xFLAG | pCambia1300_VAL2_HiFi_F | ggacgagctcggtaccATGGAGTCAATAAAGGTTTG |
|  | VAL2_3xFLAG_HiFi_R | gccctggcgcGTTCACAGGATCATGAGC |
|  | VAL2_3xFLAG_HiFi_F | tcctgtgaacGCGCCAGGGCCCTGGTTC |
|  | 3xFLAG_pCambia1300_HiFi_R | atacgaacgaaagctctgcagTTACTTATCGTCATCGTCCTTGTAATCG |
| pCAMBIA1300-35Spro-nVAL2-3xFLAG | pCambia1300_VAL2_HiFi_F | ggacgagctcggtaccATGGAGTCAATAAAGGTTTG |
|  | VAL2-N_3xFLAG_HiFi_R | gccctggcgcATTCAAGCTGTTGGAAAAC |
|  | VAL2-N_3xFLAG_HiFi_F | cagcttgaatGCGCCAGGGCCCTGGTTC |
|  | 3xFLAG_pCambia1300_HiFi_R | atacgaacgaaagctctgcagTTACTTATCGTCATCGTCCTTGTAATCG |
| pCAMBIA1300-35Spro-eYFP-NLS-3FLAG | pCambia1300_eYFP_HiFi_F | ggacgagctcggtaccATGAGCAAGGGCGAGGAG |
|  | eYFP_NLS_3xFLAG_HiFi_R | gccctggcgcTACCTTTCTCTTCTTTTTTGGATCCG |
|  | eYFP_NLS_3xFLAG_HiFi_F | gagaaaggtaGCGCCAGGGCCCTGGTTC |
|  | 3xFLAG_pCambia1300_HiFi_R | atacgaacgaaagctctgcagTTACTTATCGTCATCGTCCTTGTAATCG |

**Genotyping and qPCR primers**

| **primer name** | **primer sequence (5’ - 3’)** |
| --- | --- |
| *Atsdr4l-4*_Atsdr4l-5_GT_F | ACCTTCCCTACCTCATCAC |
| *Atsdr4l-4*_GT_R | TTATGGGCTGCGGTGATATG |
| *Atsdr4l-5*_GT_R | ACTAGAAGAACCGCATGACG |
| At1g27461.1_SDR4_q2F | CTCGTGAACTCCGCTTACAA |
| At1g27461.1_SDR4_q2R | CACTTCTCCACAGATCCTCTTC |
| At1g49240.1_ACT8_q2F | CCCGAGCAGCATGAAGATTA |
| At1g49240.1_ACT8_q2R | CTGAGGGAAGCAAGGATAGAAC |

## References

**Amselem J, Cornut G, Choisne N, Alaux M, Alfama-Depauw F, Jamilloux V, Maumus F, Letellier T, Luyten I, Pommier C, et al** (2019) RepetDB: a unified resource for transposable element references. Mob DNA **10**: 6

**Bailey TL, Johnson J, Grant CE, Noble WS** (2015) The MEME Suite. Nucleic Acids Res **43**: W39–W49

**Carroll TS, Liang Z, Salama R, Stark R, De Santiago I** (2014) Impact of artifact removal on ChIP quality metrics in ChIP-seq and ChIP-exo data. Front Genet. doi: 10.3389/fgene.2014.00075

**Chen S, Zhou Y, Chen Y, Gu J** (2018) fastp: an ultra-fast all-in-one FASTQ preprocessor. Bioinformatics **34**: i884–i890

**Clough SJ, Bent AF** (1998) Floral dip: a simplified method forAgrobacterium-mediated transformation ofArabidopsis thaliana: Floral dip transformation of Arabidopsis. Plant J **16**: 735–743

**Jumper J, Evans R, Pritzel A, Green T, Figurnov M, Ronneberger O, Tunyasuvunakool K, Bates R, Žídek A, Potapenko A, et al** (2021) Highly accurate protein structure prediction with AlphaFold. Nature **596**: 583–589

**Lamesch P, Berardini TZ, Li D, Swarbreck D, Wilks C, Sasidharan R, Muller R, Dreher K, Alexander DL, Garcia-Hernandez M, et al** (2012) The Arabidopsis Information Resource (TAIR): improved gene annotation and new tools. Nucleic Acids Res **40**: D1202–D1210

**Langmead B, Salzberg SL** (2012) Fast gapped-read alignment with Bowtie 2. Nat Methods **9**: 357–359

**Li H, Handsaker B, Wysoker A, Fennell T, Ruan J, Homer N, Marth G, Abecasis G, Durbin R, 1000 Genome Project Data Processing Subgroup** (2009) The Sequence Alignment/Map format and SAMtools. Bioinformatics **25**: 2078–2079

**Li Q, Brown JB, Huang H, Bickel PJ** (2011) Measuring reproducibility of high-throughput experiments. Ann Appl Stat. doi: 10.1214/11-AOAS466

**Love MI, Huber W, Anders S** (2014) Moderated estimation of fold change and dispersion for RNA-seq data with DESeq2. Genome Biol **15**: 550

**Machanick P, Bailey TL** (2011) MEME-ChIP: motif analysis of large DNA datasets. Bioinformatics **27**: 1696–1697

**Mirdita M, Schütze K, Moriwaki Y, Heo L, Ovchinnikov S, Steinegger M** (2022) ColabFold: making protein folding accessible to all. Nat Methods **19**: 679–682

**Pagès H** (2021) BSgenome: Software infrastructure for efficient representation of full genomes and their SNPs. doi: 10.18129/B9.bioc.BSgenome

**Ramírez F, Ryan DP, Grüning B, Bhardwaj V, Kilpert F, Richter AS, Heyne S, Dündar F, Manke T** (2016) deepTools2: a next generation web server for deep-sequencing data analysis. Nucleic Acids Res **44**: W160–W165

**Robinson JT, Thorvaldsdóttir H, Winckler W, Guttman M, Lander ES, Getz G, Mesirov JP** (2011) Integrative genomics viewer. Nat Biotechnol **29**: 24–26

**Rory S, Brown G** (2011) DiffBind: differential binding analysis of ChIP-Seq peak data.

**RStudio Team** (2020) RStudio: Integrated Development for R.

**Song L, Huang S -s. C, Wise A, Castanon R, Nery JR, Chen H, Watanabe M, Thomas J, Bar-Joseph Z, Ecker JR** (2016a) A transcription factor hierarchy defines an environmental stress response network. Science **354**: aag1550–aag1550

**Song L, Koga Y, Ecker JR** (2016b) Profiling of Transcription Factor Binding Events by Chromatin Immunoprecipitation Sequencing (ChIP-seq): Chromatin Immunoprecipitation Sequencing (ChIP-seq). Curr Protoc Plant Biol **1**: 293–306

**The R Development Core Team** (2020) R: A Language and Environment for Statistical Computing.

**Wang Z-P, Xing H-L, Dong L, Zhang H-Y, Han C-Y, Wang X-C, Chen Q-J** (2015) Egg cell-specific promoter-controlled CRISPR/Cas9 efficiently generates homozygous mutants for multiple target genes in Arabidopsis in a single generation. Genome Biol **16**: 144

**Wickham H, Averick M, Bryan J, Chang W, McGowan LD, François R, Grolemund G, Hayes A, Henry L, Hester J, et al** (2019) Welcome to the Tidyverse. J Open Source Softw **4**: 1686

**Wu T, Alizadeh M, Lu B, Cheng J, Hoy R, Bu M, Laqua E, Tang D, He J, Go D, et al** (2022) The transcriptional co‐repressor SEED DORMANCY 4‐LIKE (AtSDR4L) promotes the embryonic‐to‐vegetative transition in *Arabidopsis thaliana*. J Integr Plant Biol **64**: 2075–2096

**Yuan L, Song X, Zhang L, Yu Y, Liang Z, Lei Y, Ruan J, Tan B, Liu J, Li C** (2021) The transcriptional repressors VAL1 and VAL2 recruit PRC2 for genome-wide Polycomb silencing in *Arabidopsis*. Nucleic Acids Res **49**: 98–113

**Zhang Y, Liu T, Meyer CA, Eeckhoute J, Johnson DS, Bernstein BE, Nussbaum C, Myers RM, Brown M, Li W, et al** (2008) Model-based Analysis of ChIP-Seq (MACS). Genome Biol **9**: R137

**The R Development Core Team** (2020) R: A Language and Environment for Statistical Computing.
